# Down but not out in posterior cingulate cortex: Deactivation yet functional coupling with prefrontal cortex during demanding semantic cognition

**DOI:** 10.1016/j.neuroimage.2016.07.060

**Published:** 2016-11-01

**Authors:** Katya Krieger-Redwood, Elizabeth Jefferies, Theodoros Karapanagiotidis, Robert Seymour, Adonany Nunes, Jit Wei Aaron Ang, Vierra Majernikova, Giovanna Mollo, Jonathan Smallwood

**Affiliations:** Department of Psychology/York Neuroimaging Centre, University of York, Heslington, York, United Kingdom

**Keywords:** Posterior cingulate cortex, Dorsolateral prefrontal cortex, Semantic control, Executive, Rest, Connectivity

## Abstract

The posterior cingulate cortex (pCC) often deactivates during complex tasks, and at rest is often only weakly correlated with regions that play a general role in the control of cognition. These observations led to the hypothesis that pCC contributes to automatic aspects of memory retrieval and cognition. Recent work, however, has suggested that the pCC may support both automatic and controlled forms of memory processing and may do so by changing its communication with regions that are important in the control of cognition across multiple domains. The current study examined these alternative views by characterising the functional coupling of the pCC in easy semantic decisions (based on strong global associations) and in harder semantic tasks (matching words on the basis of specific non-dominant features). Increasingly difficult semantic decisions led to the expected pattern of deactivation in the pCC; however, psychophysiological interaction analysis revealed that, under these conditions, the pCC exhibited greater connectivity with dorsolateral prefrontal cortex (PFC), relative to both easier semantic decisions and to a period of rest. In a second experiment using different participants, we found that functional coupling at rest between the pCC and the same region of dorsolateral PFC was stronger for participants who were more efficient at semantic tasks when assessed in a subsequent laboratory session. Thus, although overall levels of activity in the pCC are reduced during external tasks, this region may show greater coupling with executive control regions when information is retrieved from memory in a goal-directed manner.

## Highlights

• We characterized the role of the posterior cingulate cortex (pCC) in tasks requiring automatic and controlled semantic memory processing
• Connectivity of posterior cingulate cortex (pCC) with dorsolateral prefrontal cortex (PFC) increased when performing demanding semantic tasks
• Stronger functional coupling of pCC with dorsolateral PFC at rest predicted more efficient semantic task performance
• Despite deactivating during demanding tasks, the pCC increases communication with prefrontal regions, suggesting a role in controlled cognition

## Introduction

The posterior cingulate cortex (pCC) is thought to play a central role in cognition but its specific contribution remains unclear (Leech et al., 2012). In conjunction with the medial prefrontal cortex, the pCC is a key hub in the so-called default mode network (DMN; Buckner et al., 2008, Raichle, 2015), a large-scale network initially defined through its tendency to deactivate during external tasks (Raichle et al., 2001) and the pattern of reduced correlation with task-positive systems that it shows at rest (Fox et al., 2006). Initial work on the role of the pCC in cognition suggested that it is important when information from memory comes automatically to mind, including during thoughts about the future (Schacter et al., 2007), easy judgments about global semantic associations (Jackson et al., 2016) and during naturally occurring stimulus-independent thought (Mason et al., 2007, Stawarczyk et al., 2011). These high levels of activity in the pCC when cognition is automatically generated from memory has led to the pCC being contrasted with neural systems that play a general role in cognitive control, such as the multiple-demand network (MDN; Duncan, 2010).

If the contribution of the pCC to cognition is primarily through automatic memory retrieval, it should be less engaged when information from memory must be retrieved in a goal related manner. Although this would explain why the pCC often deactivates in complex tasks, studies have found that even when it does this, it can remain functionally coupled to executive control regions in the MDN (Leech et al., 2012). Other studies have found evidence of co-activation between the pCC and regions of the MDN when making personal plans (Gerlach et al., 2011), generating creative solutions to problems (for a review see: Beaty et al., 2016) or in demanding working memory tasks (Konishi et al., 2015, Spreng et al., 2014, Vatansever et al., 2015).

Together these studies provide converging evidence that cooperation between the pCC and regions of the MDN occurs when information from memory must be directed towards a particular goal. The current study tests this hypothesis in the context of semantic cognition, using tasks with a well-documented reliance on both memory representations and control (Jefferies, 2013, Noonan et al., 2013, Whitney et al., 2011a, Whitney et al., 2011b). According to component process accounts of semantic cognition, anterior regions of the temporal lobe draw together different aspects of knowledge to form amodal conceptual representations (Patterson et al., 2007), while control regions in and beyond prefrontal cortex allow these representations to be deployed in an appropriate manner with respect to the specific goals of a task (Badre et al., 2005, Noonan et al., 2013, Thompson-Schill et al., 1997). Consequently, some semantic tasks that involve matching words on the basis of dominant global associations (e.g., retrieving that salt goes with pepper) are thought to be relatively automatic, since uncontrolled spreading activation within the semantic store can uncover these links efficiently. In contrast, semantic tasks that require the retrieval of specific non-dominant aspects of knowledge (e.g., whether salt is the same colour as snow) require a greater degree of control, since retrieval must be focussed on task-relevant information and away from irrelevant yet strong conceptual links (Wagner et al., 2000, Wagner et al., 2001, Whitney et al., 2011a, Whitney et al., 2011b).

We conducted two experiments to understand the contribution of the pCC to easy (more automatic) and harder (more controlled) semantic decisions and its interaction with regions involved in cognitive control at rest and during these tasks. Prior to performing these studies we performed a meta-analysis of semantic terms using the Neurosynth meta-analytic search tool, to identify a region of the pCC involved in semantic cognition and then explored the similarities between its functional coupling at rest and this meta-analytic map, as well as regions that are known to be important in cognitive control. Experiment 1 used task-based functional Magnetic Resonance Imaging (fMRI) to characterise the functional coupling of this region of pCC in easy semantic decisions (based on strong global associations) and in harder semantic tasks (matching words on the basis of specific non-dominant features). We used a psychophysiological interaction analysis (PPI) to explore how the coupling of the pCC with other regions of cortex changed with the difficulty of the semantic decisions being made. Experiment 2 used resting state fMRI to examine whether the functional connectivity of the pCC at rest varied as a function of participants' efficiency in semantic decisions. These experiments provide converging evidence that the pCC reorganises its functional connectivity when semantic information from memory is deployed in a controlled way in the service of task, and that patterns of similar connectivity at rest predict the efficiency with which these decisions can be made.

## Method

This study was approved by the University of York Neuroimaging Centre and by the Department of Psychology ethics committees. All volunteers provided informed written consent.

### Design

The aim of this study was to identify how functional communication changes between the pCC and regions of cortex involved in executive control when participants make complex semantic decisions. We selected an area of pCC from a recently published cortical parcellation of pCC (Bzdok et al., 2015) that overlapped with a meta-analytic map downloaded from Neurosynth (search term: “semantic”; 844 contributing studies; http://www.neurosynth.org/analyses/terms/) to use as a seed in our connectivity analyses. The study had two main stages: (1) task-based fMRI, and (2) resting-state functional data correlations with lab-based behavioural testing.

*Stage 1* involved collecting data from participants who performed semantic judgements ‘online’ while functional brain data were acquired. We analysed this data in two ways: we examined functional contrasts of the tasks over implicit baseline (i.e., rest) and contrasts comparing easy and harder semantic decisions; and secondly we performed psychophysiological interactions (PPI) to characterise the functional connectivity of the pCC region to other brain areas during the tasks, to examine how this changed with increased executive semantic load.

*Stage 2* focussed on resting state functional connectivity in independent data sets. Again, this involved two steps: First, we examined the correlation between RS connectivity of pCC and performance on the same semantic judgements as in Stage 1, in a new group of participants. We investigated whether regions that were more connected to pCC on-line during semantic tasks also showed stronger resting-state connectivity to pCC in participants who were good at semantic judgements outside the scanner. Secondly, in a large-scale publically-available dataset, we examined the resting-state connectivity of the brain region identified in this analysis – i.e., voxels that were more coupled to pCC in semantic tasks (PPI result) and in the resting state behavioural regression.

### Participants

All participants were native English speakers, right-handed, had normal/corrected vision and had no history of psychiatric or neurological illness. In this study we analysed resting state data from four different groups of participants. This allowed us to examine independent cohorts of participants to avoid concerns of bias through ‘double-dipping’. The first three cohorts were acquired at the University of York. Cohort 1 included RS data from 39 participants (27 females; mean age = 22.7, SD = 3.2). We used these data to create RS maps of pCC connectivity for comparison with the MDN and a meta-analytic map of semantic cognition. Cohort 2 included task-based fMRI data and resting state (RS) from 20 volunteers (11 females; mean age = 23.2, SD = 4.4). These data were used to perform task-based PPI and to compare the RS network of the pCC with task-based connectivity measures *from the same participants*. Cohort 3 included 48 participants who completed a RS scan and behavioural tasks in the lab in a subsequent session; two participants were removed from this sample due to poor co-registration of RS scans and a further two participants were removed due to being behavioural outliers (leaving n = 44 for analysis; 32 females; mean age = 20, SD = 1.7). The data acquired from these participants were used to assess whether the coupling of pCC networks at rest have implications for performance on semantic judgement tasks. We did not have task-based fMRI for these participants. We also utilised a publically available data set of 141 participants (Cohort 4, Mean Age = 37, SD = 13.9, 102 females) from the Nathan Kline Institute (NKI; Nooner et al., 2012; see Gorgolewski et al. (2014) to establish the pattern of functional connectivity at rest of the region more connected to pCC during semantic cognition (by seeding a mask defined by the task- and resting-state connectivity analyses described above).

### Tasks

For both the on line scanning session and the behavioural testing session, three semantic judgement tasks were used that ranged in difficulty from easy to hard (Fig. 2A). Two of these tasks involved judgements about global semantic associations, where the probe and target words were either strongly related (easiest trials, benefitting from automatic spreading activation; e.g., salt – pepper, diary, land; Collins and Loftus, 1975) or more weakly related (more difficult decisions; e.g., salt – grain, diary, land; Badre et al., 2005). The third task, feature selection, with the highest executive demands, required participants to match the probe to the target based on a specific feature (colour, texture, shape, size), while also suppressing the strongly associated word presented in the same trial (e.g., colour: salt – snow, pepper, diary; Badre et al., 2005, Whitney et al., 2012). Participants were instructed as to which feature to attend to at the beginning of a block of trials. In each task, a probe word appeared above three possible targets for selection, and participants were required to press a button to indicate which of the three choices matched the probe.

### Stimuli

All stimuli were nouns and were taken from Whitney et al. (2012; originally based on Badre et al. 2005). Trials in all three conditions were matched in frequency across both experiments (log frequency from Celex, Baayen et al., 1995): Feature selection, Experiment 1: *M* = 1.39, *SD* = 0.44; Experiment 2: *M* = 1.39, *SD* = 0.39. Weak association, Experiment 1: *M* = 1.53, *SD* = 0.47; Experiment 2: *M* = 1.46, *SD* = 0.45. Strong association, Experiment 1: *M* = 1.53, *SD* = 0.48; Experiment 2: *M* = 1.54, *SD* = 0.44. There were no differences in lexical frequency between conditions: Experiment 1: F(2, 78) = 1.16, *p* = 0.32; Experiment 2: F(2, 126) = 2.33, *p* = 0.1. Syllable length was also matched across conditions in both experiments: Feature selection, Experiment 1: *M* = 1.58, *SD* = 0.34; Experiment 2: *M* = 1.58, *SD* = 0.34. Weak association, Experiment 1: *M* = 1.5, *SD* = 0.33; Experiment 2: *M* = 1.51, *SD* = 0.35. Strong association, Experiment 1: *M* = 1.46, *SD* = 0.29; Experiment 2: *M* = 1.5, *SD* = 0.3. There were no differences in length between conditions: Experiment 1: F(2, 78) = 1.55, *p* = 0.22; Experiment 2: F(2, 126) = 1.03, *p* = 0.36.

### Image acquisition

#### MRI acquisition

Structural and functional data were acquired using a 3T GE HDx Excite MRI scanner utilising an eight-channel phased array head coil (GE) tuned to 127.4 MHz, at the York Neuroimaging Centre, University of York. Structural MRI acquisition in all participants was based on a T1-weighted 3D fast spoiled gradient echo sequence (TR = 7.8 ms, TE = minimum full, flip angle 20°, matrix size = 256 × 256, 176 slices, voxel size = 1.13 × 1.13 × 1 mm). Task-based and resting-state activity was recorded from the whole brain using single-shot 2D gradient-echo echo planar imaging (EPI) with a flip angle = 90°, matrix size = 64 × 64, voxel size = 3 mm^3^, and field of view (FOV) = 192 mm^2^. Other scan parameters slightly varied for task-based fMRI (TR = 3000 ms, TE = 19 ms, 60 slices, 260 volumes) and resting-state fMRI (Cohort 1: TR = 2000 ms, TE = minimum full, 32 slices with 0.5 mm gap, 210 volumes; Cohort 2: TR = 3000 ms, TE = 29 ms, 60 slices, 180 volumes; Cohort 3: TR = 3000 ms, TE = minimum full, 60 slices, 180 volumes). An intermediary FLAIR scan with the same orientation as the functional scans was collected to improve the co-registration between subject-specific structural and functional scans. Parameters of the independent (NKI)/Rockland Enhanced Sample are described in detail by Gorgolewski et al. (2014) and Smallwood et al. (2016).

### Data pre-processing and analysis

*a) Task-based fMRI*. Analyses were conducted at the first and higher level using FSL-FEAT version 5.98, part of FSL (FMRIB's Software Library, www.fmrib.ox.ac.uk/fsl) (Jenkinson et al., 2012, Smith et al., 2004, Woolrich et al., 2009). Pre-processing included slice timing correction using Fourier-space time-series phase-shifting (interleaved), motion correction using MCFLIRT (Jenkinson et al., 2002), high-pass temporal filtering (Gaussian-weighted least-squares straight line fitting, with sigma = 35 s), brain extraction (Smith, 2002), linear co-registration to the corresponding T1-weighted image followed by linear co-registration to MNI152 standard space (Jenkinson and Smith, 2001), spatial smoothing using a Gaussian kernel with full-width-half-maximum (FWHM) of 6 mm and grand-mean intensity normalisation of the entire 4D dataset by a single multiplicative factor.

Pre-processed time series data were modelled using a general linear model correcting for local autocorrelation (Woolrich et al., 2001). We used a block design – the linear model included the three experimental conditions (block start times and durations for each task type). Five contrasts were defined: individual conditions > rest (feature selection, weak association, strong association); feature selection > strong association and feature selection > weak association. Our analysis focussed on the comparison of executively demanding feature selection vs. relatively automatic strong associations. All analyses were cluster corrected using a *z*-statistic threshold of 3.1 to define contiguous clusters (Worsley, 2001) and then corrected for multiple comparisons at *p* < 0.005 FWE. We also performed the same analysis using a more liberal correction for multiple comparisons (*p* < 0.05 FWE) which yielded almost identical results and so we report the more conservative threshold in the paper and upload the unthresholded maps onto Neuorvault.

We extracted the time-course from the pCC mask to look for psychophysiological interactions (PPI; O'Reilly et al., 2012) between the pCC and other brain regions that differ according to task load (i.e., an interaction between feature > strong associations and the functional coupling of the pCC with other brain areas). The extracted time-course of pCC and the interaction were included in a GLM model as explanatory variables (at the lower level, for each participant and each task individually). These were then submitted to a group level analysis, as with the functional data, with the same contrasts and cluster forming threshold.

*b) Resting-state fMRI*. Pre-processing steps were as for task fMRI, except for the Gaussian low pass temporal filtering, with sigma = 2.8 s. We extracted the time series from masks of pCC (analyses using York Cohorts 1, 2 and 3) and dorsolateral prefrontal cortex (dorsolateral PFC; analysis using NKI data; mask derived from the overlap of the functional (n = 20) and RS (n = 20) analyses) and used these as explanatory variables in connectivity analyses at the single subject level. In each analysis, we entered 11 nuisance regressors; the top five principal components extracted from white matter (WM) and cerebrospinal fluid (CSF) masks based on the CompCor method (Behzadi et al., 2007) and six head motion parameters. WM and CSF masks were generated from each individual's high resolution structural image (Zhang et al., 2001). No global signal regression was performed, following the method implemented in Murphy et al. (2009). At the group-level, analyses were carried out using FMRIB's Local Analysis of Mixed Effects (FLAME1), using a cluster correction (*p* < 0.05), with a *z*-statistic threshold of 2.3 to define contiguous clusters at the group level. Analysis three included behavioural regressors (demeaned z-scored efficiency scores: (accuracy z-scored) – (RT z-score)) in the FLAME model to evaluate the connectivity of pCC to areas within the DMN in relation to semantic task performance. The connectivity map resulting from seeding the dorsolateral PFC was uploaded to Neurovault to use the image decoder (http://www.neurosynth.org/decode/), allowing us to extract key terms associated with the positive connectivity map of this region.

### Region of interest selection and mask creation

The binarised pCC seed mask was taken from a previously published cortical parcellation of pCC (Bzdok et al., 2015; parcellation subregion 2). This mask fell within the semantic map downloaded from Neurosynth (search term: “semantic”; 844 contributing studies; http://www.neurosynth.org/analyses/terms/) and was the only pCC subregion that Bzdok et al. (2015) reported as being consistently associated with language processing.

All maps generated in this study are freely available at the following URL at Neurovault: http://neurovault.org/collections/1268/.

## Results

The aim of this study was to identify how functional communication changes between the pCC and regions of the cortex involved in executive control when participants make demanding semantic decisions. Our analysis has four stages. First, we use a meta-analytic approach to identify a region of the pCC that is important in semantic cognition. Second, we examine how this region of pCC changes its functional coupling with regions in the dorsolateral PFC when participants make more difficult semantic decisions. Third, we show that this pattern of coupling at rest predicts the effectiveness with which participants make semantic decisions. Finally we used a functional connectivity analysis and meta-analytic decoding to characterise the region of dorsolateral PFC identified through the prior stage of our analysis.

### Identification of the region of pCC that overlaps with semantic networks

**Fig. 1:**
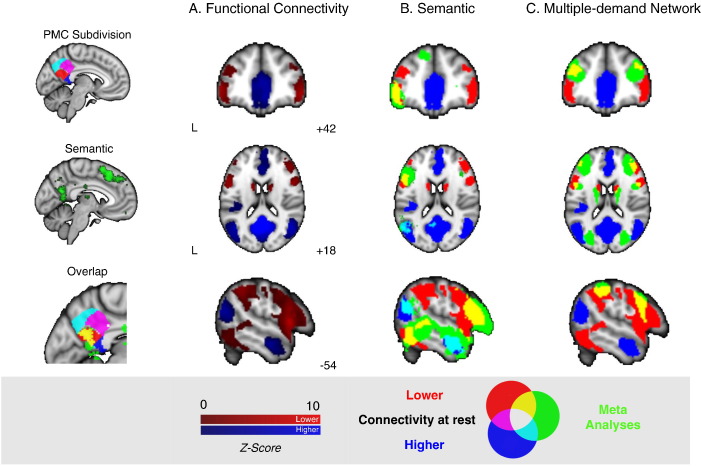
Left-hand column shows overlap (yellow) of the pCC subdivisions with a semantic meta-analytic map (green) derived from Neurosynth (using ‘semantic’ as a search term). (A): Positive (blue) and negative (red) functional connectivity of pCC at rest (cluster correction, *Z* > 2.3, *p* < 0.05), and the overlap of these positive and negative networks with (B) semantic control (Noonan et al., 2013) and (C) the multiple-demand network (MDN; Duncan, 2010) shown in yellow (overlap of low connectivity at rest and semantic control/MDN) and cyan (overlap of high connectivity at rest and semantic control/MDN). Maps in Panels B & C are displayed with a fully saturated colour map to maximise the visibility of the regions of overlap.

The pCC is a complex region of cortex with heterogeneous patterns of functional connectivity (Bzdok et al., 2015, Leech et al., 2011, Margulies et al., 2009). The first stage in our analysis used a meta-analytic approach to identify the region of the pCC that is important in semantic processing. We selected four regions identified through a data-driven parcellation of the pCC (Bzdok et al., 2015) and compared these to a meta-analytic map generated for the search term semantic using Neurosynth (Yarkoni et al., 2011). One region of pCC overlapped with the meta-analytic map for the term semantics (see sub panel Fig. 1). This region also corresponds to the posterior core of the DMN (Andrews-Hanna et al., 2010).

To understand how this region of pCC communicates with other cortical regions at rest, we examined its functional connectivity at rest in Cohort 1 (see Fig. 1A, Table 1). This pCC region demonstrated a pattern of connectivity that corresponded to the canonical DMN: relatively strong coupling was observed in bilateral medial prefrontal cortex, lateral temporal lobes, and angular gyrus. Regions exhibiting relatively weak levels of connectivity included parts of lateral frontal (e.g., IFG), precentral gyrus, supramarginal gyrus (SMG) and inferior temporal areas (ITG extending into inferior lateral occipital cortex).

We compared these spatial maps of pCC connectivity to both the meta-analysis of semantic tasks from Neurosynth (Fig. 1B), and with the spatial distribution of the MDN that supports cognitive control (Fig. 1C; Fedorenko et al., 2013). Comparison with the Neurosynth meta-analysis of the term semantic revealed that regions that are either strongly correlated with pCC (e.g., angular gyrus or the anterior temporal lobe) and those that are relatively weakly correlated (e.g., left prefrontal cortex) overlap with those important for semantic cognition. Brain regions that showed relatively weak connectivity with the pCC at rest overlapped with those generally recruited during difficult tasks (e.g. lateral prefrontal cortex or the inferior parietal sulcus).

### Experiment 1: behaviour of pCC during difficult semantic decision-making

Having identified a region of the pCC that is engaged in semantic processing yet anti-correlated with regions involved in the general control of cognition at rest, Experiment 1 explored how this region changes its connectivity in difficult semantic tasks in a sample of 20 healthy participants. Consistent with expectations, we observed clear differences in the behavioural data: performance was poorer for feature judgements than for judgements about strong associations (t(19) = 17.8, *p* ≤ 0.001) and weak associations (t(19) = 11.04, *p* ≤ 0.001). Performance was also poorer for weak associations than strong associations (t(19) = − 6.6, *p* ≤ 0.001; efficiency scores).

**Fig. 2:**
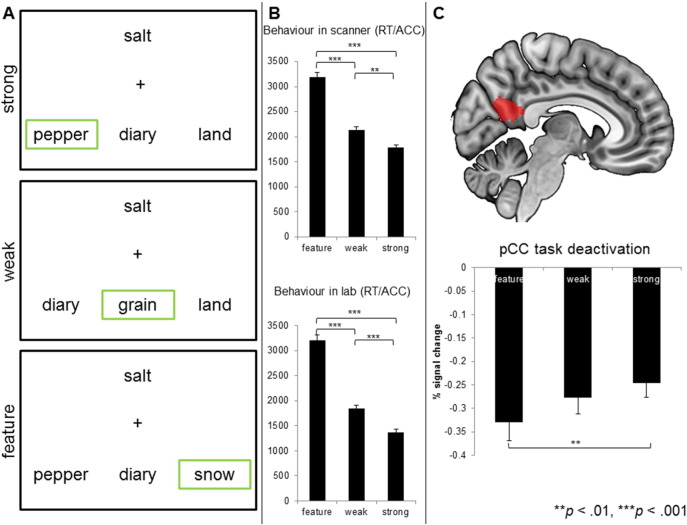
(A): task conditions for experiments 1 and 2, the target item is highlighted (green box). (B): efficiency scores (RT/ACC) for experiments 1 and 2, in milliseconds. (C): ROI analysis of percent signal change in pCC (mask shown in red). Error bars indicate standard error of the mean for both behavioural and ROI data (**p < 0.01, ***p < 0.001).

**Fig. 3:**
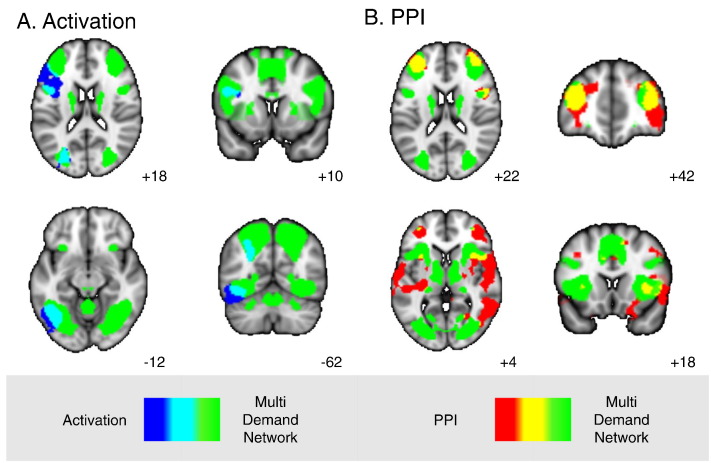
Whole brain contrasts of feature selection > strong association for functional activation (A) and PPI of pCC (B; cluster correction, *Z* > 3.1, *p* < 0.005). Overlap with Duncan's (2010) multiple-demand network is shown in cyan for the functional activation and in yellow for the PPI. These maps are displayed with a fully saturated colour map to maximise the visibility of the areas of overlap.

A region of interest analysis within the pCC mask demonstrated the expected relative deactivation as task demands increased. Greater deactivation in pCC was observed when participants made more demanding semantic decisions (Fig. 2C, feature vs. high: t(19) = − 3.45, *p* < 0.01; feature vs. low: t(19) = − 1.43, *p* > 0.1; low vs. high: t(19) = 1.12, *p* > 0.1; Bonferroni corrected). At the whole brain level, a contrast of feature selection over strong global association judgements was used to document the neural changes that occur when making more difficult semantic decisions. This is summarized in Fig. 3A (and Table 2; tasks over rest: supplementary Table 1). The feature task was associated with an increased BOLD response in left hemisphere regions including temporal occipital fusiform cortex, lateral occipital cortex and precentral gyrus extending into IFG (Table 2). These regions have been reported before in previous fMRI studies employing similar executively-demanding semantic tasks (e.g., Badre et al., 2005, Krieger-Redwood et al., 2015, Thompson-Schill et al., 1997). A comparison of these regions of activation with the MDN mask (represented in cyan) indicates that several regions activated by the feature task fell within areas commonly activated by demanding cognitive tasks beyond semantics. Together these results demonstrate that more difficult semantic decisions lead to greater deactivation in the key region of the pCC and increased BOLD activity in key regions of the MDN.

We next conducted a PPI analysis to examine whether making more difficult semantic decisions leads the pCC to change its functional connectivity with other regions of the brain. A whole brain comparison of Feature > Strong is presented in Fig. 3B (and Table 2; feature > baseline: supplementary Table 1). This revealed increased coupling between the pCC and regions of left and right frontal cortex (frontal pole, supramarginal gyrus, middle frontal gyrus) and left temporal cortex (planum temporale, parahippocampal gyrus). Several of these regions overlapped with areas of the MDN (in dorsolateral PFC bilaterally, right insula and inferior temporal gyrus/inferior lateral occipital cortex, represented in yellow).

**Fig. 4:**
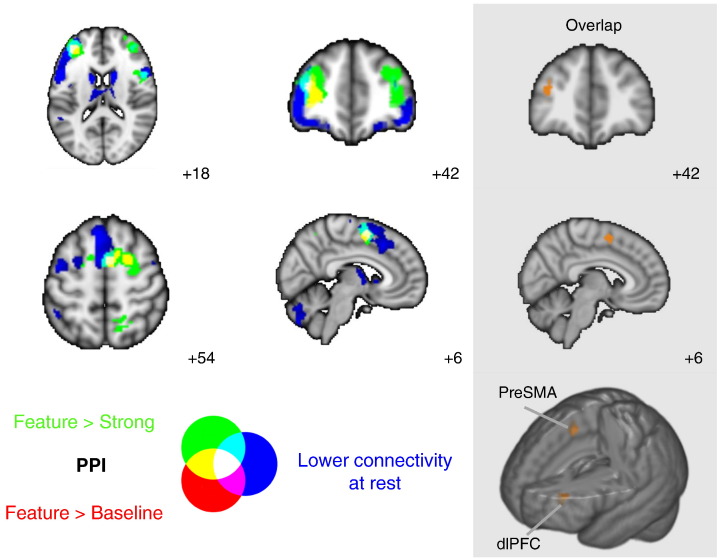
Task-based pCC functional connectivity masked by the multiple-demand network (Duncan, 2010). Overlap (white) of (i) pCC task-based functional connectivity for contrasts of feature selection > strong association (green) and feature > baseline (red) and (ii) lower resting state connectivity of pCC (blue). The grey panel displays the overlap of the pCC PPI contrasts and lower resting state connectivity revealed two clusters: one in dorsolateral prefrontal cortex (dlPFC) and one in pre-supplementary motor area (preSMA). These maps are displayed with a fully saturated colour map to maximise the visibility of the overlaps.

Next we explored whether these regions of heightened coupling between the pCC and the MDN also correspond to regions that changed their activity from rest. We masked the whole brain PPI results by the MDN, highlighting regions of cortex that are often associated with executively-demanding cognition, using a binarised mask of the MDN (Fedorenko et al., 2013). The resulting maps were then compared with the negative connectivity map of the pCC at rest from the same participants. The outcome of this analysis is presented in Fig. 4. Two regions of the MDN – the dorsolateral PFC and preSMA – increased their connectivity with the pCC during feature matching relative to both the strong association task and to rest. The specific areas of overlap are presented in the panel of Fig. 4.

### Experiment 2: coupling between pCC and the MDN at rest – implications for semantic performance

Having demonstrated that the pCC changes its pattern of connectivity during demanding semantic decision-making relative to both rest and easy semantic retrieval tasks – by increasing its coupling to dorsolateral PFC, we next examined whether the functional coupling of the pCC with the regions at rest conveys information on how effectively participants will perform on these semantic tasks. This analysis is important since it helps determine whether the strength of coupling between pCC and dorsolateral PFC is important for effective task performance, or instead reflects a pattern of neural communication that occurs when participants perceive a task to be difficult or detect errors. Using a separate sample of participants (Cohort 3; n = 44), we measured resting state brain activity and then assessed behavioural performance on the same three tasks used in Experiment 1 several days later in the laboratory. As with Experiment 1 we observed the expected differences in behavioural performance across the three conditions (feature vs. strong: t(43) = 16.27, *p* ≤ 0.001; feature vs. weak: t(43) = 13.49, *p* ≤ 0.001; weak vs. strong: t(43) = 11.79, *p* ≤ 0.001; see Fig. 2B).

**Fig. 5:**
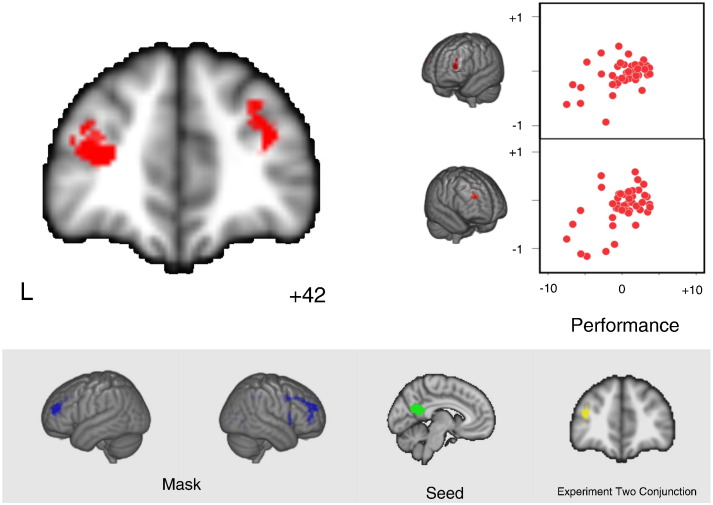
Multiple regression result of resting state functional connectivity and response efficiency on semantic tasks (cluster correction, *Z* > 2.3, *p* < 0.05; search space restricted by a mask created using the final result of experiment 1: PPI masked by MDN contrast of feature > strong; blue). The correlation of performance and resting state connectivity is shown in the right hand scatterplots. A high score on the x-axis indicates better task performance.

We conducted a multiple regression in which the independent variables were efficiency scores describing the participants' performance on the semantic tasks and the dependent variable was the whole brain connectivity of the pCC at rest. This analysis was masked with the results of the whole brain PPI map generated by the conjunction of the contrast of feature > strong in Experiment 1 and the MDN, allowing our analysis to focus on regions of the MDN that had exhibited increased functional coupling with the pCC during task states. We created a binarised mask using the thresholded statistical map derived from the PPI analysis of pCC (feature selection > strong association), masked by the MDN (for confirmation, we ran the same analysis using masks generated from a PPI analysis thresholded at *p* < 0.05, and the results were identical; therefore we report the *p* < 0.005 mask here, for consistency across the paper). We formulated contrasts to identify areas whose connectivity with the pCC predicted better or worse performance on each task, as well as average performance across tasks. The results of this analysis are presented in Fig. 5 where it can be seen that connectivity of the pCC with dorsolateral PFC was stronger for participants who on average performed better on all three tasks (individual scatter plots for each task are presented in supplementary Fig. 1). Boostrapping analysis confirmed the reliability of these correlations (Right dLPFC: r = + 0.53 95% CI = + 0.24, + 0.73; lDLPFC: r = + 0.42, 95% CI = + 0.07, + 0.67). Importantly, Experiment 2 shows that stronger coupling between the pCC and the dorsolateral PFC is high for people who will subsequently do well; therefore, this pattern of functional coupling must underlie more effective performance rather than processes which occur when task demands exceed a person's capability to perform the task.

### Characterising the connectivity and functional significance of the region in dorsolateral PFC

**Fig. 6:**
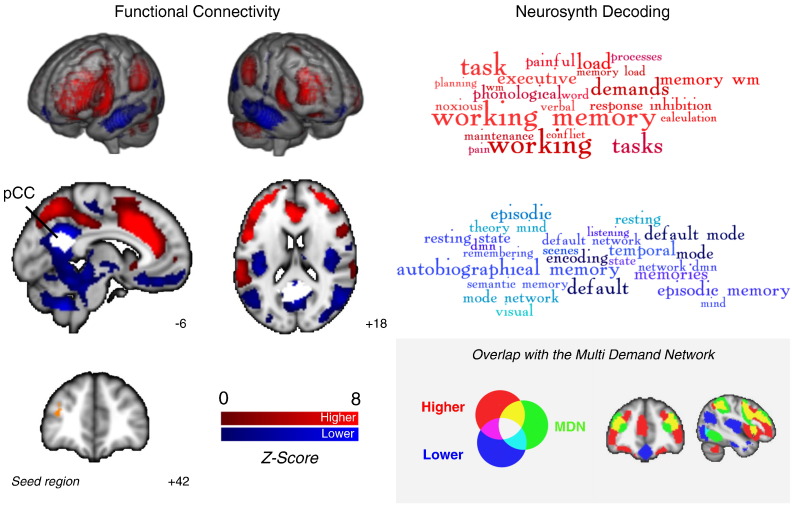
Higher (red) and lower (blue) resting state connectivity of dorsolateral prefrontal cortex (seed region: overlap of Experiments 1 and 2), and the corresponding terms derived from Neurosynth for these maps. The grey panel displays the positive connectivity map for this region overlaps with parts of the multiple-demand network (Duncan, 2010; yellow). In this panel the maps are displayed with a fully saturated colour map to maximise the visibility of the overlaps.

Next, we characterised the connectivity of the dorsolateral PFC region whose connectivity to the pCC was found to be important for effective semantic decision-making. Using a separate cohort of participants from a publicly available database (Cohort 4), we performed resting state connectivity analysis using the region of the left dorsolateral PFC that was commonly implicated in Experiments 1 and 2. A binarised mask of the dorsolateral PFC was created by taking the overlap of the MDN masked PPI analysis feature selection > strong association and feature > baseline, the negative connectivity of the pCC (Cohort 2; n = 20) and the RS behavioural regression result (Cohort 3; n = 44). This mask was used in a functional connectivity analysis. Fig. 6 confirms that this region of dorsolateral PFC was positively correlated with lateral prefrontal cortex, insula, pre-SMA and anterior parts of inferior parietal cortex – all key components of the MDN. Decoding this connectivity map using Neurosynth identified terms consistent with a role in cognitive control including “task demands”, “working memory” and “executive load”. The dorsolateral PFC region showed relatively weak correlation with lateral temporal cortex, ventromedial PFC and pCC, regions in the core DMN, an interpretation confirmed by the Neurosynth decoding results. This analysis shows that this region of the dorsolateral PFC is important in the process of cognitive control and a member of the MDN. Notably we also observed that the dorsolateral PFC showed a pattern of positive connectivity with a region of medial parietal cortex adjacent to our pCC seed. This positively-coupled region of pCC corresponded to a different region (pCC 1 and 3) of the parcellation conducted by Bzdok et al. (2015).

### Consistency across samples

**Fig. 7:**
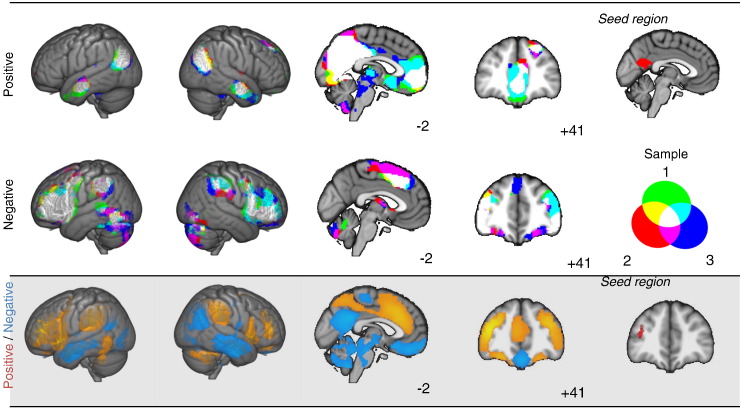
Comparison of the patterns of functional connectivity in different cohorts used in this study. The upper panel illustrates the positive and negative resting state connectivity for the pCC region studied in three cohorts of participants used in this experiment. The lower panel presents the connectivity of the dorso-lateral prefrontal seed from Cohort 4. All maps in this figure are displayed with a fully saturated colour map.

In our final analysis we consider whether the patterns of connectivity that we generated through the course of this study are consistent across the different samples. Fig. 7 presents the spatial overlap between the connectivity maps generated for pCC in Cohorts 1, 2 and 3. It can be seen that there is a high degree of overlap in both the regions showing relatively strong and weak connectivity with the seed region. In the grey panel, we also present the connectivity of the dorsolateral PFC for the purpose of visual comparison. There is also a broad degree of overlap between the regions showing stronger connectivity with the dLPFC and those showing weaker connectivity with the pCC (and vice versa). This indicates that, despite differences in the phenotypical or demographic features of the sample, there is nonetheless a high degree of consistency across the different data sets.

## Discussion

The current study investigated the contribution of the pCC to semantic cognition. Experiment 1 demonstrated that the pCC deactivates during difficult feature-matching judgements and yet shows increased functional connectivity with regions of the multiple-demand network (MDN; Duncan, 2010), in particular a region of left dorsolateral prefrontal cortex (PFC), during this task. In Experiment 2, the presence of this pattern of functional coupling at rest was predictive of being able to make semantic decisions more efficiently in a subsequent laboratory session. Thus, functional coupling between pCC and dorsolateral PFC underlies the capacity to make effective semantic decisions. Together these results show that the contribution of the pCC to semantic cognition is not limited to situations in which information from memory must be retrieved automatically. Instead, it is also implicated when information from memory is used in a controlled fashion and under these circumstances it increases its functional coupling with regions of cortex that support cognitive control.

There are a number of reasons to expect that the observed connectivity between the pCC and lateral prefrontal regions supports aspects of cognition beyond semantic cognition. First, co-recruitment of pCC and dorsolateral PFC is not limited to semantic tasks: this pattern is also observed in tasks of working memory (Konishi et al., 2015), creativity (Beaty et al., 2016) and future planning (Gerlach et al., 2011, Spreng et al., 2010). Like our feature matching semantic task, these situations share the need to use information from memory in a controlled and flexible way, in service of a specific goal. Second, the region of dorsolateral PFC that shows connectivity with the pCC is activated by a wide range of executively-demanding tasks (Fedorenko et al., 2013, Fedorenko and Thompson-Schill, 2014). Coupling of the pCC and the dorsolateral PFC therefore occurs in difficult tasks that are not exclusively semantic.

More generally, these findings help to refine our understanding of the manner in which the pCC contributes to cognition. The meta-analysis of the initial decomposition of the pCC that was the starting point of our investigation highlighted the pCC region as important for many forms of higher order thought, including language and semantic discrimination (see Bzdok et al., 2015) and we supported this conclusion through our comparison with a meta-analysis of semantic tasks using Neurosynth. Our functional study shows that the behaviour of this region of pCC may reflect the complex nature of on-going cognition, not through its overall levels of activity, but through its pattern of connectivity with other regions of cortex. Connectivity studies in humans and primates suggests that the broader pCC acts as a cortical hub that integrates activity across many brain systems (Bzdok et al., 2015, Leech et al., 2011, Margulies et al., 2009). This view of pCC function is consistent with functional and anatomical evidence showing that this broad region is strongly connected to diverse areas, including executive control sites beyond the DMN (Braga et al., 2013, Bzdok et al., 2015, Leech et al., 2012, Margulies et al., 2009). For example, Leech et al. (2011) observed spatial subdivisions within the pCC: while ventral pCC (territory similar to our seed region) showed strong connectivity to the rest of the DMN, more anterior and dorsal regions were found to couple to regions implicated in cognitive control. We recovered this distinction in our final analysis of the resting-state connectivity of the dorsolateral PFC, which showed that while ventral pCC showed relatively weak or negative connectivity, more anterior pCC was strongly coupled to this executive region. Our data show that engaging in executively-demanding semantic memory retrieval leads to changes in the coupling of the ventral pCC, so that it becomes more similar to that observed at rest in the adjacent anterior pCC region. The connectivity of the ventral pCC seed region changed to reflect ongoing task demands to a greater extent than adjacent anterior pCC, which showed this pattern even at rest (see supplementary Fig. 2). These data support the suggestion by Bzdok et al. (2015) that the pCC may show spatial subdivisions in its patterns of connectivity at rest that change during task states. We hypothesise that the pCC territory that corresponds to the core DMN may model the state of different neural systems dynamically, depending on the nature of environmental demands (Pearson et al., 2011), or possibly even the ongoing train of thought in which the participant is engaged in (Smallwood et al., 2016).

Finally, our results have important implications for understanding the relationship between neural processes that emerge during tasks and at rest. Our data builds on prior work that has shown similar patterns of co-ordinated neural functioning can occur at rest and during tasks (e.g., Smith et al., 2009), extending these findings in two important ways. First, although we found broad similarities in our two experiments in terms of their connectivity between pCC and dorsolateral PFC there is an important difference. In Experiment 1 the coupling of the pCC and dorsolateral PFC increased during tasks that were more difficult, while in Experiment 2 we found that people who performed well on semantic tasks in general showed the same pattern of connectivity at rest. Although sharing a similar spatial location for both the seed and target, these results differ in their task specificity: the resting state correlations reflect a general potential to perform well on semantic tasks, whereas the PPI results reflect the application of this process in a specific task context. It is also important to note that our analysis shows that the worst performers showed patterns of negative pCC to dlPFC connectivity, whereas better performers tended to show patterns of connectivity that were close to zero. It is possible that, at least as assessed across a period of wakeful rest, a pattern of connectivity between these two regions that is close to zero may be optimal for semantic cognition. Second, unlike prior studies that focused on the *similarities* between connectivity patterns at rest and during tasks, our results highlight the functional significance of *changes* from what is normally observed at rest. Experiment 1 shows that during complex semantic decision-making, the pCC reorganises its connectivity from patterns seen across a group of participants at rest, while Experiment 2 shows that the extent to which this pattern is present in an individual at rest reflects the efficiency with which they can make these decisions. Our results therefore illustrate that the similarities between neural processing during tasks and rest extend beyond those patterns of connectivity that are generally true at the population level. Instead they indicate that certain features of functionally-relevant neural organization emerge as deviations from the patterns that are traditionally seen at rest. It will be important in the future to test this idea through the assessment of whether the functional coupling of the pCC to regions of dlPFC is associated with periods of heightened task performance, a question that our block level PPI is unable to answer. Such investigations will help understand whether certain neural systems, such as those anchored by the pCC, influence cognition through their capacity to flexibly reorganise their coupling with other regions of cortex in line with the changing demands posed by the external environment (Pearson et al., 2011).

## Figures and Tables

**Table 1:** Connectivity of pCC seed at rest; cluster correction, *Z* > 2.3, *p* < 0.05.

	Connectivity		*Z*	x	y	z	Voxels
Higher	L	Precuneus	12	–8	–58	26	16829
	R	Posterior cingulate gyrus	12	4	–50	16	
	L	Precuneus	11.4	–6	–66	24	
	R	Precuneus	11.4	8	–64	22	
	R	Posterior cingulate gyrus	11.4	4	–48	26	
	L	Precuneus	10.9	–8	–64	14	
	R	Frontal pole	7.7	0	60	–4	6062
	L	Frontal medial cortex	6.73	–6	52	–12	
	R	Anterior cingulate gyrus	6.69	6	40	8	
	R	Paracingulate gyrus	6.37	4	48	4	
	R	Paracingulate gyrus	6.35	8	42	–4	
	R	Paracingulate gyrus	6.17	10	44	0	
	R	Middle temporal gyrus	6.36	60	–16	–16	1156
	R	Middle temporal gyrus	5.91	60	–6	–22	
	R	Temporal pole	4.46	40	14	–34	
	R	Middle temporal gyrus	3.8	50	0	–26	
	L	Middle temporal gyrus	5.69	–60	–8	–26	865
	L	Middle temporal gyrus	5.62	–54	–14	–20	
	L	Middle temporal gyrus	5.51	–62	–14	–18	
	L	Temporal pole	3.31	–44	12	–36	
Lower	L	Inferior frontal gyrus (pars opercularis)	7.64	–52	10	8	8969
	L	Frontal pole	6.24	–48	40	0	
	L	Temporal pole	6.01	–52	14	–8	
	L	Insular cortex	5.68	–40	18	–2	
	L	Angular gyrus	5.42	–52	–52	50	
	L	Frontal pole	5.38	–44	36	–14	
	R	Inferior frontal gyrus (pars opercularis)	6.61	52	12	10	5990
	R	Frontal pole	6.39	50	42	–6	
	R	Frontal pole/inferior frontal gyrus (pars triangularis)	6.38	48	34	–4	
	R	Precentral gyrus/inferior frontal gyrus (pars opercularis)	5.93	54	10	18	
	R	Frontal orbital cortex	5.91	36	28	–4	
	R	Temporal pole	5.83	52	16	–10	
	R	Cerebellum	6.62	34	–68	–36	2095
	R	Cerebellum	5.45	30	–68	–32	
	R	Cerebellum	5.32	34	–82	–30	
	R	Cerebellum	4.38	20	–74	–28	
	R	Cerebellum	4.27	12	–80	–22	
	R	Inferior lateral occipital cortex	4.2	44	–78	–16	
	L	Cerebellum	4.4	–12	–80	–32	1857
	L	Posterior inferior temporal gyrus	4.33	–48	–36	–18	
	L	posterior superior temporal gyrus	4.14	–66	–40	8	
	L	Occipital fusiform gyrus	4.05	–38	–66	–24	
	L	Cerebellum	4	–26	–68	–34	
	L	Cerebellum	3.78	–40	–66	–30	
	L	Paracingulate gyrus	5.52	–4	16	44	1523
	L	Juxtapositional lobule/supplementary motor cortex	5.35	–6	2	54	
	R	Juxtapositional lobule/supplementary motor cortex	5.22	4	2	54	
	R	Juxtapositional lobule/supplementary motor cortex	5.05	8	8	50	
	L	Paracingulate gyrus	4.63	–8	10	52	
	R	Paracingulate gyrus	3.51	8	26	40	
	R	Posterior supramarginal gyrus	5.33	60	–40	46	1254
	R	Anterior supramarginal gyrus	4.75	58	–28	44	
	R	Posterior supramarginal gyrus	4.46	40	–38	40	
	R	Anterior Supramarginal Gyrus	4.45	62	–28	46	
	R	Posterior supramarginal gyrus	4.42	48	–38	54	
	R	Posterior supramarginal gyrus	4.34	48	–40	50	

**Table 2:** Functional and PPI clusters; cluster correction, *Z* > 3.1, *p* < 0.005.

	Activation peaks		*Z*	x	y	z	Voxels
Functional feature-strong	L	Temporal occipital fusiform cortex	5.54	–38	–52	–18	1283
	L	Inferior temporal gyrus, temporooccipital part	5.16	–46	–52	–14	
	L	Temporal occipital fusiform cortex	5.14	–44	–60	–14	
	L	Temporal occipital fusiform cortex	4.84	–36	–58	–12	
	L	Temporal occipital fusiform cortex	4.57	–36	–64	–14	
	L	Inferior temporal gyrus, temporooccipital part	4.37	–54	–60	–22	
	L	Lateral occipital cortex, superior division	4.99	–28	–66	38	556
	L	Lateral occipital cortex, superior division	3.84	–22	–70	54	
	L	Precentral gyrus	4.56	–42	0	28	521
	L	Precentral gyrus	3.55	–46	–4	38	
	L	Frontal operculum	3.19	–34	18	16	
PPI feature-strong	R	Supramarginal gyrus	6.01	56	–42	38	18355
	R	Supramarginal gyrus, posterior division	5.98	64	–38	32	
	R	Frontal pole	5.7	40	44	10	
	L	Precuneus cortex	5.6	–6	–46	50	
	R	Precuneus cortex	5.43	4	–42	46	
	R	Juxtapositional lobule cortex/supplementary motor cortex	5.43	12	4	60	
	L	Planum temporale	6.28	–54	–32	10	5341
	L	Supramarginal gyrus, posterior division	5.88	–54	–42	22	
	L	White matter/putamen/insula	5.71	–30	–24	4	
	L	Heschl's Gyrus (H1&H2)	5.59	–48	–26	8	
	L	Parahippocampal gyrus	5.39	–24	–28	–18	
	L	Supramarginal gyrus, posterior division	5.37	–60	–46	24	
	L	Frontal pole	5.86	–34	42	18	1611
	L	Frontal pole	5.46	–36	42	6	
	L	Frontal pole	5.31	–34	44	26	
	L	Middle frontal gyrus	4.54	–38	20	38	
	L	Middle frontal gyrus	4.48	–34	28	40	
	L	Middle frontal gyrus	4.15	–36	32	32	
PPI MDN mask feature	L	Frontal pole	4.48	–34	42	6	364
	L	Frontal pole	4.18	–36	42	16	
	L	Frontal pole	3.62	–32	46	26	
	L	Frontal pole	3.25	–44	48	18	
	R	Juxtapositional lobule cortex/supplementary motor cortex	4.69	10	4	60	309
	R	Superior frontal gyrus	4.02	22	6	56	
	R	Juxtapositional lobule cortex/supplementary motor cortex	4.02	6	2	52	
	R	Cerebral white matter	3.19	18	0	46	
PPI MDN mask feature-strong	R	Frontal pole	5.7	40	44	10	2548
	R	Frontal pole	5.17	34	36	28	
	R	Precentral gyrus	5.16	46	–2	40	
	R	Frontal pole	4.96	44	46	–2	
	R	Middle frontal gyrus	4.96	36	32	36	
	R	Middle frontal gyrus	4.76	42	24	42	
	L	Frontal pole	5.86	–34	42	18	1365
	L	Frontal pole	5.46	–36	42	6	
	L	Frontal pole	5.31	–34	44	26	
	L	Middle frontal gyrus	4.46	–36	28	38	
	L	Middle frontal gyrus	4.39	–40	20	38	
	L	Middle frontal gyrus	4.15	–36	32	32	
	R	Juxtapositional lobule cortex/supplementary motor cortex	5.43	12	4	60	1149
	R	Juxtapositional lobule cortex/supplementary motor cortex	5.17	8	2	50	
	R	Superior frontal gyrus	5.13	26	4	54	
	L	Superior frontal gyrus	5.03	–14	4	60	
	R	Middle frontal gyrus	4.17	30	–4	54	
	R	Precentral gyrus	3.91	26	–8	58	
	R	Supramarginal gyrus, posterior division	6.01	56	–42	38	786
	R	Angular gyrus	5.21	42	–52	42	
	R	Supramarginal gyrus, posterior division	4.25	32	–44	34	
	R	Angular gyrus	3.92	46	–44	30	
	R	Superior parietal lobule	3.82	28	–46	46	
	R	Lateral occipital cortex, superior division	3.49	34	–62	38	
	R	Precuneous	5.28	14	–70	40	327
	R	Lateral occipital cortex, superior division	4.51	20	–62	48	
	R	Precuneus	4.17	14	–70	48	
	R	Precuneus	4.07	16	–52	54	
	R	Superior parietal lobule	3.71	24	–56	52	
	R	Lateral occipital cortex, superior division	3.58	12	–60	56	
	R	Lateral occipital cortex, inferior division	4.42	44	–62	–2	310
	R	Lateral occipital cortex, inferior division	3.56	46	–64	–16	
	R	Temporal occipital fusiform cortex	3.55	44	–58	–20	
	R	Lateral occipital cortex, inferior division	3.51	46	–76	6	
	R	Inferior temporal gyrus, temporooccipital part	3.4	56	–50	–10	
	R	Lateral occipital cortex, inferior division	3.38	44	–76	12	

## Appendix A Supplementary data

Supplementary material. Image 1
