# Supplementary material for: Down but not out in posterior cingulate cortex: Deactivation yet functional coupling with prefrontal cortex during demanding semantic cognition
Source: Neuroimage. 2016 Nov 1;141:366–77. doi: 10.1016/j.neuroimage.2016.07.060 (PMC5035136; doi:10.1016/j.neuroimage.2016.07.060)
Supplement: Supplementary file 1 — Supplementary material. [file mmc1.pdf]

Supplementary Materials

We conducted a multiple regression in which the independent variables were efficiency scores describing the participants' performance on the semantic tasks and the dependent variable was the whole brain connectivity of the pCC at rest. We created a binarised mask using the thresholded statistical map derived from the PPI analysis of pCC (feature selection > strong association), masked by the MDN. We formulated contrasts to identify areas whose connectivity with the pCC predicted better or worse performance on each task, as well as on average. The results of this analysis demonstrated that connectivity of the pCC with dorsolateral PFC was stronger for participants who on average performed better on all three tasks. Here, we present individual scatter plots for each task and resting state connectivity of pCC and dorsolateral PFC, extracted using featquery (Supplementary Figure 1).

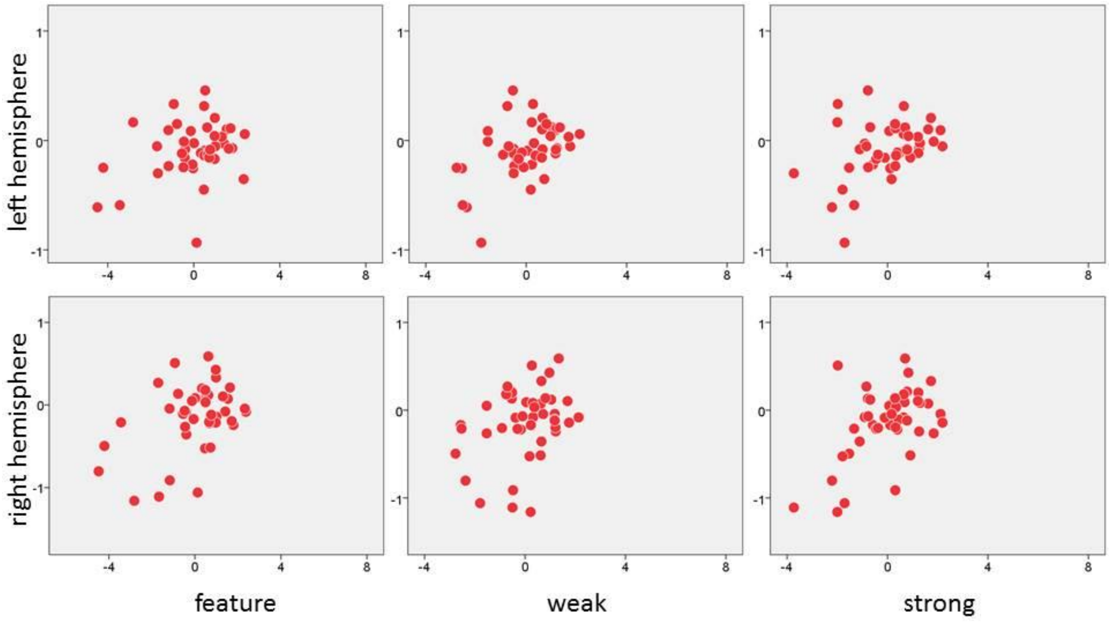

Supplementary Figure 1. The correlation of performance on each task and resting state connectivity of pCC and dorsolateral PFC.

We used featquery to interrogate the pCC-dorsolateral PFC connectivity profile for each task, across all four pCC regions identified in Bzdok et al. (2015). To do this, we conducted a PPI analysis (please refer to the main paper for PPI method) using each pCC mask from Bzdok et al. (2015), and interrogated the resulting data using a binarised mask of dorsolateral PFC (Supplementary Figure 2). A binarised mask of the dorsolateral PFC was created by taking the overlap of the MDN masked PPI analysis feature selection > strong association and feature > baseline and the negative connectivity of the pCC (Cohort 2; n = 20).

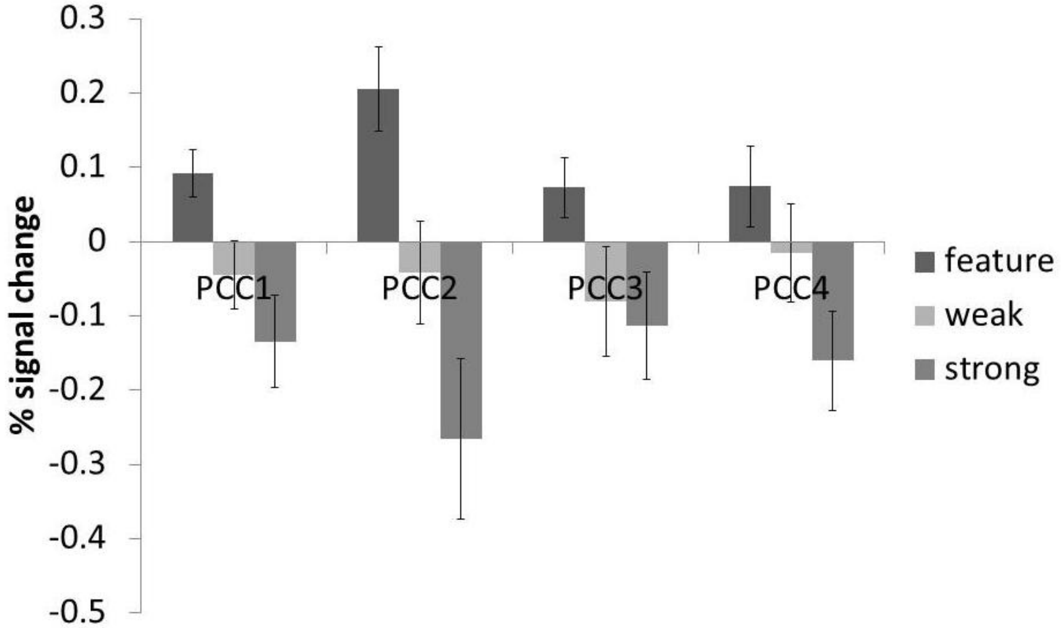

Supplementary Figure 2. ROI analysis of PPI percent signal change in dorsolateral PFC from pCC seeds (seeds taken from parcellation of Bzdok et al., 2015). Error bars indicate standard error of the mean.

The analysis of our task-based fMRI (and PPI analysis) included five contrasts: individual conditions > rest (feature selection, weak association, strong association); feature selection > strong association and feature selection > weak association. Our analysis focussed on the comparison of executively demanding feature selection vs. relatively automatic strong associations. For reference, we have reported the contrast (activation and PPI) for feature selection > weak association in Supplementary Figure 3, and also included feature selection > strong association for easy comparison across these contrasts (i.e., feature selection vs. controlled and automatic semantic association judgements). For further reference, we have also included Supplementary Table 1, which includes the results from the individual conditions > rest (feature selection, weak association, strong association). Supplementary Table 1 also includes the PPI result for feature > rest. All analyses were cluster corrected using a *z*-statistic threshold of 3.1 to define contiguous clusters (Worsley, 2001) and then corrected for multiple comparisons at $p < .005$ FWE.

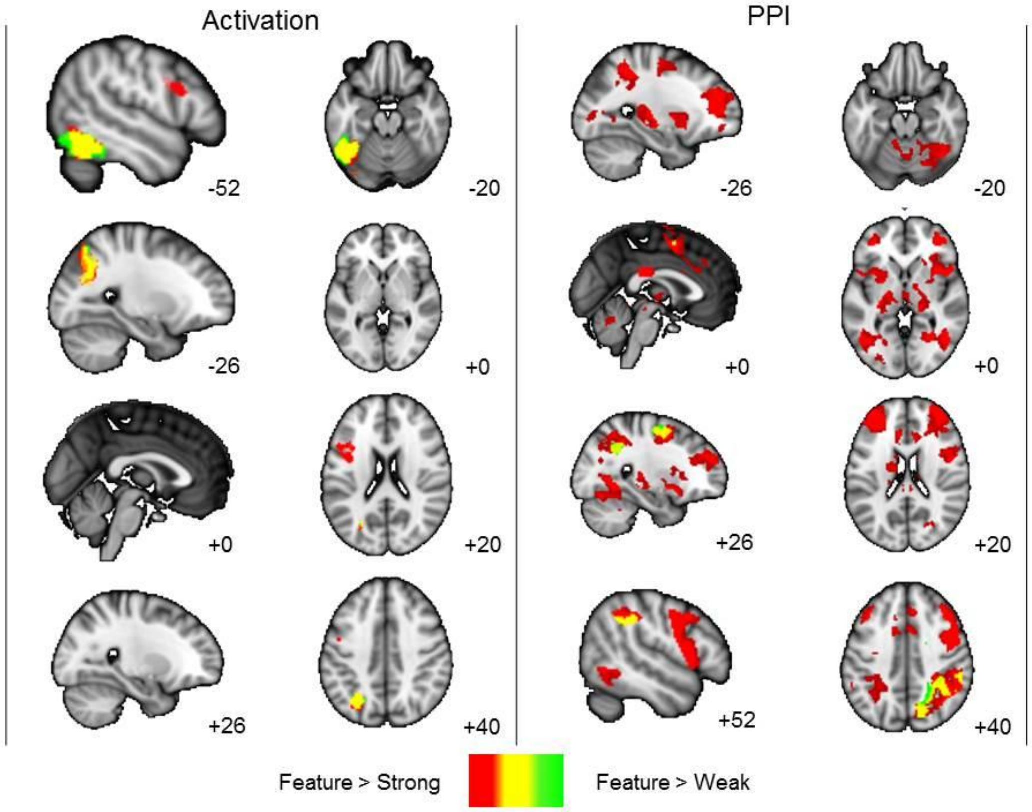

Supplementary Figure 3. Whole brain contrasts of feature selection > strong association and feature selection > weak association for functional activation and PPI of pCC (cluster correction, $Z > 3.1$, $p < .005$).

Table S1: Results of whole brain PPI of Feature > Baseline

		Activation peaks	*Z*	x	y	z	Voxels
Functional feature selection	**L**	**Precentral Gyrus**	**7.59**	**-42**	**0**	**28**	**4994**
	L	Inferior Frontal Gyrus (pars triangularis)	7.45	-52	30	18	
	L	Frontal Pole	7.29	-48	38	10	
	L	Inferior Frontal Gyrus (pars triangularis)	7.21	-50	36	14	
	L	Inferior Frontal Gyrus (pars opercularis)	5.99	-38	20	20	
	L	Insular Cortex	5.5	-30	22	-2	
	**L**	**Inferior Lateral Occipital Cortex**	**8.16**	**-44**	**-68**	**-10**	**3321**
	L	Inferior Temporal Gyrus (temporooccipital)	7.87	-46	-52	-14	
	L	Inferior Temporal Gyrus (temporooccipital)	7.71	-46	-58	-14	
	L	Posterior Temporal Fusiform Cortex	6.86	-30	-18	-36	
	L	Temporal Occipital Fusiform Cortex	6.29	-38	-52	-18	
	L	Temporal Occipital Fusiform Cortex	4.43	-24	-40	-22	
	**L**	**Anterior Supramarginal Gyrus**	**7.23**	**-52**	**-30**	**34**	**3249**
	L	Superior Lateral Occipital Cortex	7.18	-28	-68	36	
	L	Posterior Supramarginal Gyrus	7.17	-40	-42	38	
	L	Superior Lateral Occipital Cortex	6.52	-30	-64	44	
	L	Anterior Supramarginal Gyrus	6.22	-60	-28	30	
	L	Superior Lateral Occipital Cortex	5.15	-22	-70	54	
	**L**	**Paracingulate Gyrus**	**6.99**	**-4**	**12**	**48**	**849**
	L	Superior Frontal Gyrus	4.02	-4	34	46	
	L	Paracingulate Gyrus	3.99	-6	28	40	
	**L**	**Precentral Gyrus**	**5.62**	**-30**	**-8**	**50**	**738**
	L	Superior Frontal Gyrus	5.47	-20	-6	50	
	L	Middle Frontal Gyrus	4.58	-24	4	44	
	L	Middle Frontal Gyrus	4.14	-28	6	52	
	**R**	**Inferior Lateral Occipital Cortex**	**5.4**	**50**	**-82**	**0**	**680**
	R	Occipital Fusiform Gyrus	5.26	42	-64	-14	
	R	Inferior Lateral Occipital Cortex	4.45	46	-70	-6	
	R	Occipital Pole	3.34	36	-98	-12	

	R	Inferior Lateral Occipital Cortex	3.3	44	-88	12	
	**R**	**Cerebellum**	**5.83**	**34**	**-72**	**-48**	**481**
	R	Cerebellum	5.62	34	-68	-42	
	R	Cerebellum	4.69	32	-70	-32	
Functional weak association	**L**	**Inferior Frontal Gyrus (pars triangularis)**	**5.65**	**-50**	**30**	**18**	**933**
	L	Inferior Frontal Gyrus (pars triangularis)	5.01	-50	36	8	
	L	Precentral Gyrus	4.8	-42	-2	28	
	L	Precentral Gyrus	4.24	-50	4	22	
	L	Central Opercular Cortex	4.12	-38	-2	22	
	L	Inferior Frontal Gyrus (pars triangularis)	3.49	-36	22	14	
	**L**	**Anterior Supramarginal Gyrus**	**5.64**	**-52**	**-30**	**34**	**774**
	L	Posterior Supramarginal Gyrus	5.46	-38	-42	38	
	L	Anterior Supramarginal Gyrus	4.72	-44	-34	36	
	L	Postcentral Gyrus	4.44	-58	-22	22	
	L	**Inferior Lateral Occipital Cortex**	**5.02**	**-48**	**-68**	**-2**	**403**
	L	Inferior Lateral Occipital Cortex	3.98	-54	-78	4	
	L	Inferior Lateral Occipital Cortex	3.84	-52	-82	2	
	L	Inferior Lateral Occipital Cortex	3.68	-52	-82	-4	
	L	**Paracingulate Gyrus**	**6.25**	**-6**	**12**	**48**	**387**
Functional strong association	**L**	**Anterior Supramarginal Gyrus**	**5.93**	**-52**	**-30**	**34**	**1071**
	L	Anterior Supramarginal Gyrus	5.4	-38	-40	38	
	L	Anterior Supramarginal Gyrus	4.88	-58	-24	24	
PPI feature	**R**	**Posterior Supramarginal Gyrus**	**5.34**	**64**	**-38**	**32**	**856**
	R	Posterior Supramarginal Gyrus	5.02	56	-40	38	
	R	Posterior Supramarginal Gyrus	4.84	58	-36	38	
	R	Planum Temporale	4.34	64	-32	20	
	R	Angular Gyrus	4.09	52	-44	30	
	R	Angular Gyrus	4.04	52	-52	36	
	**R**	**Precuneous**	**4.48**	**4**	**-44**	**46**	**556**
	R	Posterior Cingulate Gyrus	4.21	4	-24	38	
	L	Precuneous	4.01	-2	-44	46	
	L	Precuneous	3.94	-6	-46	50	
	L	Precuneous	3.9	-12	-56	50	
	L	Posterior Cingulate Gyrus	3.71	-4	-22	36	
	**L**	**Planum Temporale**	**4.93**	**-54**	**-32**	**10**	**523**
	L	Posterior Supramarginal Gyrus	4.91	-54	-42	22	
	L	Posterior Supramarginal Gyrus	4.37	-56	-48	34	
	L	Posterior Supramarginal Gyrus	4.31	-60	-46	24	

L	Heschl's Gyrus (H1 & H2)	4.31	-50	-24	8	
L	Heschl's Gyrus (H1 & H2)	4.29	-46	-26	8	
**L**	**Frontal Pole**	**4.48**	**-34**	**42**	**6**	**368**
L	Frontal Pole	4.18	-36	42	16	
L	Frontal Pole	3.62	-32	46	26	
L	Frontal Pole	3.25	-44	48	18	
**R**	**Juxtapositional Lobule Cortex/Supplementary Motor Cortex**	**4.69**	**10**	**4**	**60**	**331**
R	Superior Frontal Gyrus	4.02	22	6	56	
R	Juxtapositional Lobule Cortex/Supplementary Motor Cortex	4.02	6	2	52	
R	White matter	3.19	18	0	46	
**R**	**Temporooccipital Middle Temporal Gyrus**	**4.12**	**62**	**-44**	**-2**	**328**
R	Temporooccipital Middle Temporal Gyrus	4.11	62	-46	-6	
R	Temporooccipital Middle Temporal Gyrus	3.98	68	-48	-4	
R	Temporooccipital Middle Temporal Gyrus	3.96	64	-54	-2	
R	Temporooccipital Middle Temporal Gyrus	3.79	60	-52	8	
R	Temporooccipital Middle Temporal Gyrus	3.5	70	-54	-4	

Supplementary Table 1; cluster correction, $Z > 3.1$, $p < .005$.
